# Space flight associated changes in astronauts’ plasma‐derived small extracellular vesicle microRNA: Biomarker identification

**DOI:** 10.1002/ctm2.845

**Published:** 2022-06-02

**Authors:** David Goukassian, Arsen Arakelyan, Agnieszka Brojakowska, Malik Bisserier, Siras Hakobyan, Lahouaria Hadri, Amit Kumar Rai, Angela Evans, Aimy Sebastian, May Truongcao, Carolina Gonzalez, Anamika Bajpai, Zhongjian Cheng, Praveen Kumar Dubey, Sankar Addya, Paul Mills, Kenneth Walsh, Raj Kishore, Matt Coleman, Venkata Naga Srikanth Garikipati

**Affiliations:** ^1^ Cardiovascular Research Institute Icahn School of Medicine at Mount Sinai New York New York USA; ^2^ Bioinformatics Group Institute of Molecular Biology, NAS RA Yerevan Armenia; ^3^ Department of Bioengineering, Bioinformatics and Molecular Biology Russian‐Armenian University Yerevan Armenia; ^4^ Armenian Bioinformatics Institute Yerevan Armenia; ^5^ Department of Emergency Medicine The Ohio State University Wexner Medical Center Columbus Ohio USA; ^6^ Department of Radiation Oncology University of California Davis Sacramento California USA; ^7^ Lawrence Livermore National Laboratory Livermore California USA; ^8^ Center for Translational Medicine Temple University School of Medicine Philadelphia Pennsylvania USA; ^9^ Department of Biomedical Engineering The University of Alabama at Birmingham Birmingham Alabama USA; ^10^ Thomas Jefferson University Philadelphia Pennsylvania USA; ^11^ Integrative Health and Mind‐Body Biomarker Laboratory University of San Diego San Diego California USA; ^12^ University of Virginia School of Medicine Charlottesville Virginia USA; ^13^ Dorothy M. Davis Heart Lung and Research Institute The Ohio State University Wexner Medical Center Columbus Ohio USA

Dear Editor,

This pilot study suggests relatively short (median 12 days long) low‐Earth orbit (LEO) spaceflight induces changes in circulating plasma small extracellular vesicle (sEV) microRNA expression. Normalization of small RNA sequencing (sRNAseq) data and quantitative polymerase chain reaction (qPCR) validation confirmed miR‐4732‐3p is significantly upregulated up to 3 days post‐landing, and enrichment analysis suggests this miRNA is expressed in various central nervous system tissues and hematopoietic cells and may be linked to different organ disorders.

During spaceflight, astronauts are exposed to numerous stressors (i.e. microgravity and ionizing radiation)^1^ that are linked to various harmful health effects, including cardiovascular (CV), neurodegenerative diseases, and cancers.^2^, ^3^ However, the molecular basis of spaceflight‐induced pathophysiological changes remains limited. Few studies have suggested LEO spaceflight promotes physiologic stress as noted by increased reactivation of Epstein Barr virus, elevated urine catecholamine and cortisol levels, and increased circulating cell‐free mitochondrial DNA and cytokines.^1^, ^4^, ^5^, ^6^, ^7^ Interestingly, NASA's Twin study also noted unique miRNA signatures (miR‐125, miR‐16, and let‐7a) in peripheral blood mononuclear cells following year‐long LEO spaceflight.^8^ Additionally, emerging evidence shows that sEVs carry cell‐specific RNA signatures that can regulate stress responses.^9^ Thus, changes in sEVs content may serve as potential predictive biomarkers of health and/or subclinical disease. Therefore, we sought to determine whether the spaceflight environment can induce alterations in sEV miRNA content.

We performed sRNAseq using sEVs isolated from deidentified plasma collected 10 days before launch (L‐10), the day of landing (R‐0), and 3 days post‐landing (R+3) from 14 astronauts who flew various space Shuttle missions between 1998–2001 (Figure 1A). This study was approved by NASA and the Icahn School of Medicine at Mount Sinai's IRBs (STUDY00000075 and HSM19‐00367, respectively). Nanosight analysis revealed that the concentration of sEVs was reduced at R+3 but the size remained unchanged (Figure 1B,C). sEV content was further characterized using an exosome‐specific antibody array confirming the preponderance of exosomal proteins (Figure 1D). The sRNAseq dataset revealed plasma sEVs were enriched with various RNA species (Figure S1A); however, the current study focused on miRNA. Batch effect assessment of variability of raw miRNA counts showed strong batch effects associated with inter‐astronaut variability of miRNA expression (Figure S1B–D). Further, the portrayal of miRNA transcriptome landscapes using self‐organizing maps confirmed the considerable interindividual variability at baseline and at both post‐flight time points (Figure 1E).

**FIGURE 1:**
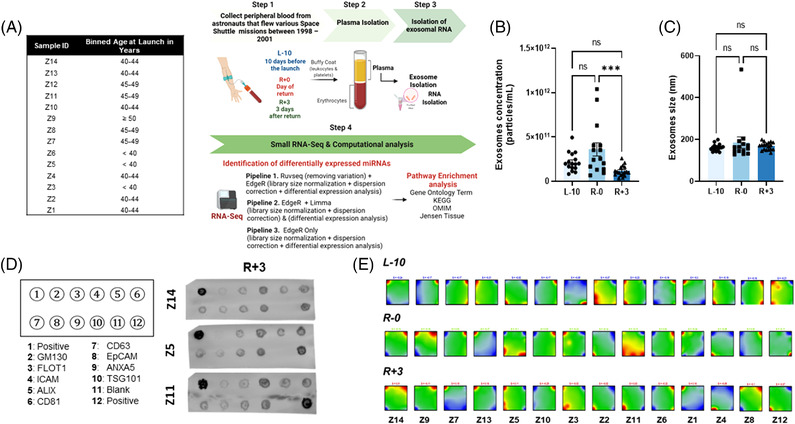
Preparation of peripheral blood small extracellular vesicle (sEV) RNA for sequencing. (A) Schematic representation of experimental design and bioinformatics pipeline. The table shows non‐attributable demographic information, binned ages, of 14 astronauts who flew space Shuttle missions between 1998–2001. Peripheral blood was isolated from astronauts at three different time points: 10 days before launch (L‐10), day of return from mission (R‐0), and 3 days after return (R+3). sEVs were isolated from plasma, and sEV‐derived total RNA was processed for small RNA sequencing (sRNAseq). Differentially expressed microRNA (miRNAs) were identified using three computational pipelines: RUVseq+EdgeR, EdgeR+Limma, and EdgeR only. For the identified miRNA, pathway enrichment analysis was performed using Gene Ontology (GO) Term, 2021 Kyoto Encyclopedia of Genes and Genomes (KEGG), Online Mendelian Inheritance in Man (OMIM), and Jensen Tissue databases. (B, C) Characterization of exosomes by concentration and size using Nanosignt analysis. (D) Exosome isolation was further validated using an exosome‐specific antibody array (exosome markers: CD63, CD81, ALIX, FLOT1, ICAM1, EpCam, ANXA5, and TSG101). Cis‐Golgi marker GM130 monitors any cellular contamination in exosome isolation, and a positive control spot was derived from human serum exosomes. (E) Self‐organizing maps (SOM) transcriptomic portrayal projects high dimensional transcript expression data onto a 2D grid map. Each map expresses a transcriptome profile for each astronaut at L‐10, R‐0, and R+3 and is characterized by red‐blue spots that reflect up‐and downregulated miRNAs

To determine alterations in miRNA expression, we utilized three different normalization methods (edgeR only, edgeR‐limma, and RUVseq‐edgeR), which identified five overlapping differentially regulated miRNAs at R‐0 compared to L‐10 (Figure 2A,D,E). Of these, four were upregulated (hsa‐miR‐424‐5p, hsa‐miR‐140‐5p, hsa‐miR‐361‐5p, hsa‐miR‐26b‐5p) and one downregulated (hsa‐miR‐363‐3p). Analyses of predicted subcellular localization revealed that most miRNAs are expressed in the cytoplasm, followed by mitochondria, nucleus, EVs, free in circulation, and exosomes (Figure 2B). Further, we identified six overlapping differentially expressed miRNAs at R+3 compared to L‐10, of which five were downregulated (hsa‐miR‐20b‐5p, hsa‐miR‐363‐3p, hsa‐miR‐4732‐3p, hsa‐miR‐5480‐3p, hsa‐miR‐627‐5p) and one was upregulated (hsa‐miR‐483‐5p) (Figure 2C,F,G). We did not detect any significant localization predictions for these six miRNAs.

**FIGURE 2:**
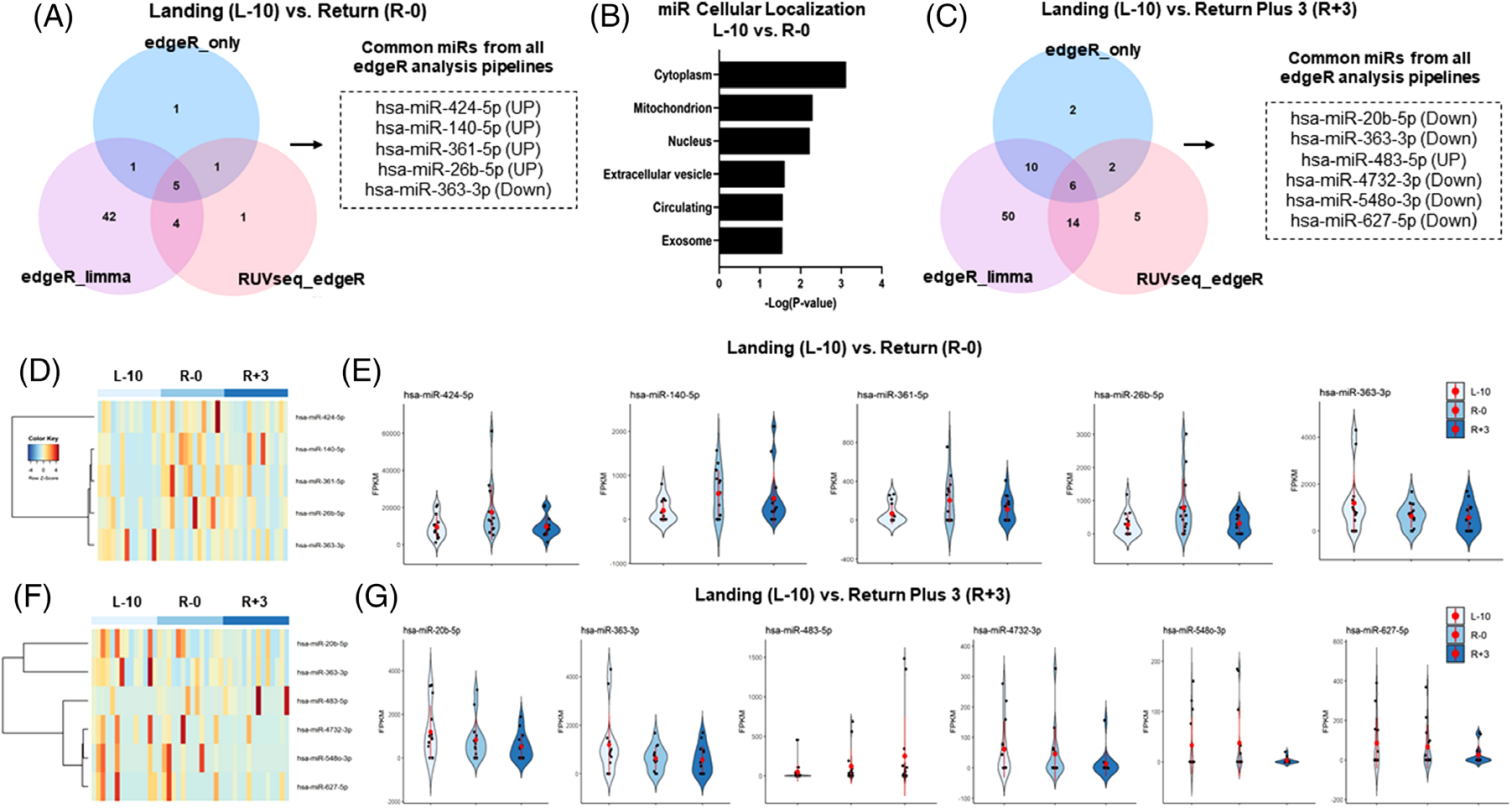
Expression profile of dysregulated microRNAs (miRNAs) after spaceflight. (A) Venn Diagram showing overlapping differentially expressed miRNA between three computational pipelines (RUVseq+edgeR, edgeR+limma, and edgeR only) when comparing small extracellular vesicle (sEV) miRNA transcriptomes between baseline (L‐10) and day of landing (R‐0). (B) Cellular localization of identified miRNA for L‐10 versus R‐0 comparison using miRNA Enrichment Analysis and Annotation Tool (miEAA) with RNALocate database. (C) Venn Diagram showing overlapping differentially expressed miRNA between three computational pipelines (RUVseq+edgeR, edgeR+limma and edgeR only) when comparing sEV miRNA transcriptomes between L‐10 and 3 days post return from mission (R+3). (D) Heatmap displaying differentially expressed miRNAs identified between the day of landing (R‐0) and baseline (L‐10) for each sample in the sRNAseq dataset. Each row represents one miRNA, and each column represents each sample across all three‐time points (L‐10, R‐0, and R+3). Red and blue gradients represent up or downregulated expression, respectively. (E) Distribution of relative expression of each identified miRNA for L‐10 versus R‐0 comparison for individual astronauts across all three‐time points. (F) Heatmap analysis of differentially expressed miRNAs identified between 3 days post landing (R+3) and L‐10 for each sample in the sRNAseq dataset. Each row represents one miRNA, and each column represents each sample across all three‐time points (L‐10, R‐0 and R+3). Red and blue gradients represent up or downregulated expression, respectively. (G) Distribution of library normalized transcript counts (FPKM) of each identified significant miRNA for L‐10 vs R+3 comparison for individual astronauts across all three‐time points

To validate these sRNAseq analysis findings, we assayed the expression of nine miRNAs by qPCR (Figure 3). Only hsa‐miR‐4732‐3p was significantly upregulated at R+3 compared to L‐10 (*p* < 0.05) (Figure 3H). Notwithstanding the substantial interindividual variability, comparing the sRNAseq datasets and qPCR analysis revealed discrepancies between these two approaches. For example, hsa‐miR‐4732‐3p expression was down‐regulated at R+3 in sRNAseq compared to qPCR results. Similarly, qPCR analysis for hsa‐miR‐363‐3p, hsa‐miR‐20b‐5p, and hsa‐miR‐627‐5p appeared to be regulated in the opposite direction to sRNAseq data (Figures 2C,F,G and 3E,F,I). Of note, sRNAseq was performed using RNA isolated from sEVs of 14 astronauts, while qPCR analysis could only be performed on seven astronauts due to the exceptionally rare sample availability. Thus, out of an abundance of caution, we narrowed our focus to hsa‐miR‐4732‐3p, which displayed significant regulation at R+3 compared to L‐10 by qPCR.

**FIGURE 3:**
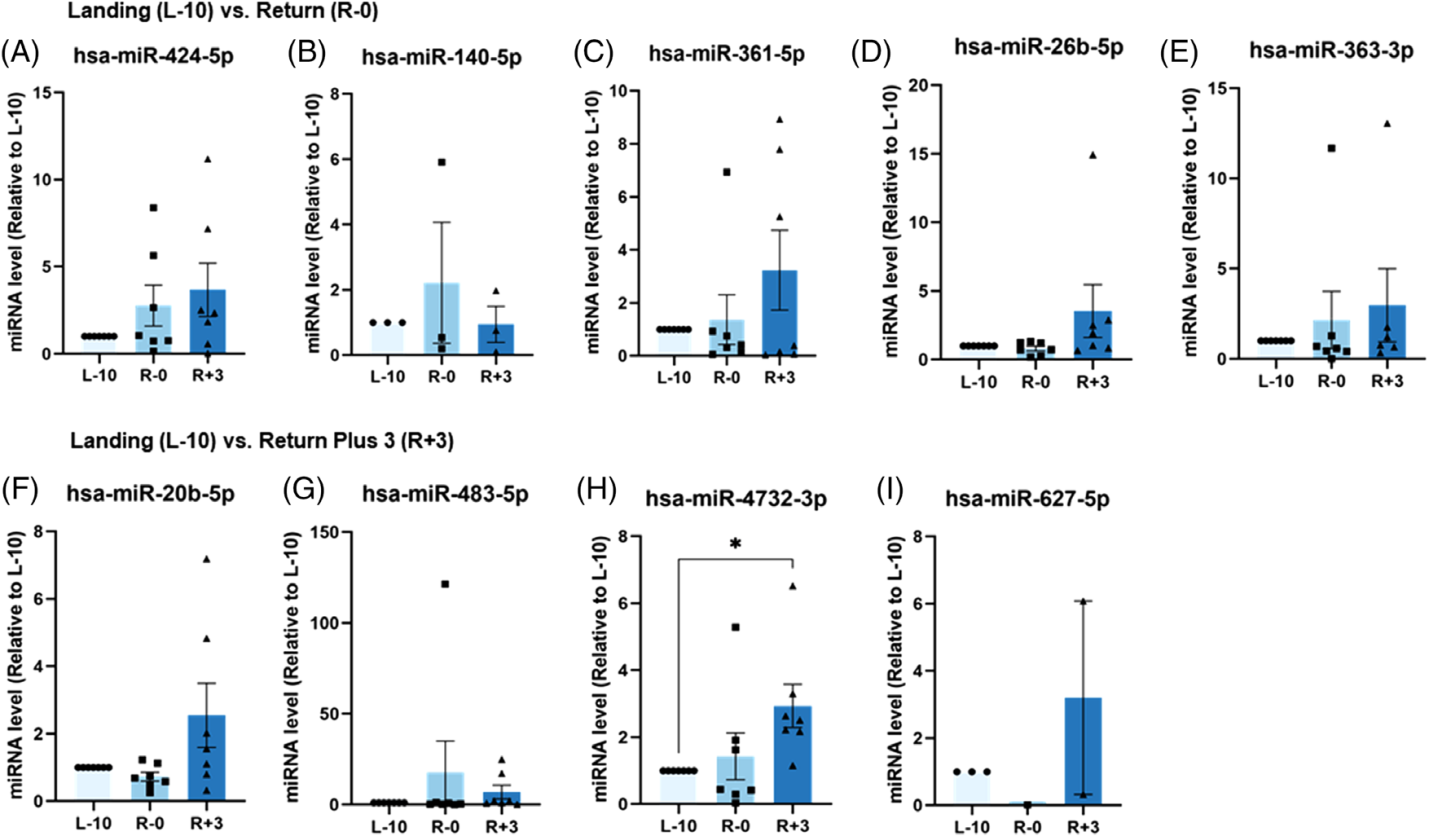
Quantitative polymerase chain reaction (qPCR) of microRNA (miRNA) dysregulated by spaceflight. (A–E) Transcript levels of miRNA were identified to be differentially expressed between baseline (L‐10) and the day of landing (R‐0). (F–I) Transcript miRNA levels differentially expressed between baseline and 3 days after return from mission (R+3). Total RNA was isolated from small extracellular vesicles (sEVs) isolated from blood plasma collected from seven astronauts at L‐10, R‐0, and R+3. Expression levels of each miRNA were measured by qPCR, and transcript counts were normalized by hsa‐miR‐423‐5p. Astronaut samples used for validation include Z14, Z13, Z11, Z9, Z7, Z6 and Z5. Each bar represents fold increases of each miRNA at R‐0 and R+3 relative to L‐10 for each astronaut. Unpaired t‐test for comparisons between means ± SEM, *p < 0.05

To better understand the potential tissues, biological pathways, and diseases that may be associated with spaceflight‐regulated hsa‐miR‐4732‐3p, we performed functional enrichment of the top 20 key hub‐nodes for this miR using MiEAA (Figure 4). Network analysis results were plotted with degree centrality and closeness centrality reflecting the node's influence on hsa‐miR‐4732‐3p (Figure 4A). Tissue enrichment analysis using the Jensen Tissues database identified adipose tissue and uterus as the most enriched, whereas the most significant number of target genes were enriched in different tissues of the central nervous system (34%), followed by the immune/lymphatic system (31%) (Figure 4B).

**FIGURE 4:**
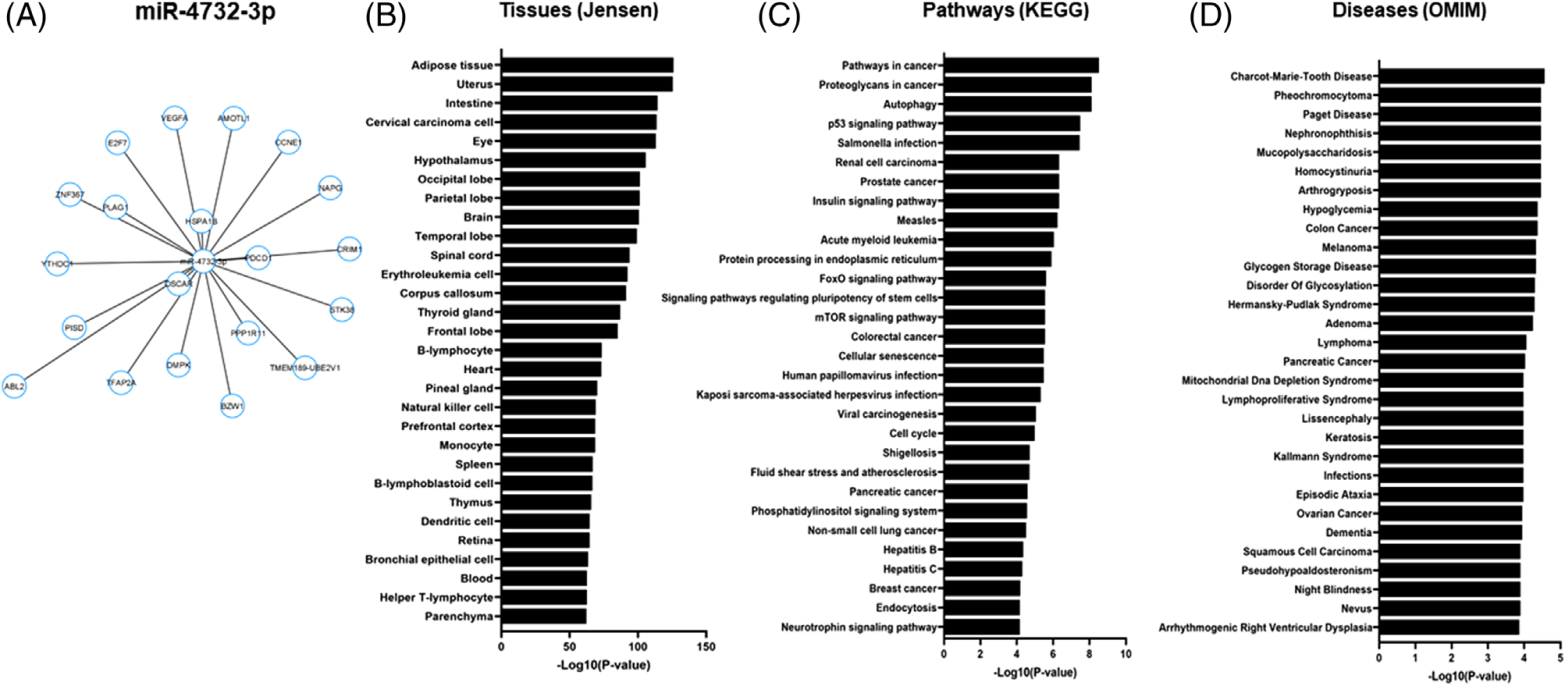
Enrichment analysis for differentially expressed miR‐4732‐3p. (A) Top 20 hub genes of network with a degree of node distribution set up with miR‐4732‐3p targets. (B) Enrichment analysis using the Jensen Tissue database was performed to identify tissue specificity of genes identified to be regulated by hsa‐miR‐4732‐3p. (C) Top 20 pathway enrichment results using the 2021 Kyoto Encyclopedia of Genes and Genomes (KEGG) database. (D) Top 20 annotated disease enrichment using Online Mendelian Inheritance In Man (OMIM)

KEGG pathway enrichment analysis revealed that the top 20 pathways identified for hsa‐miR‐4732‐3p are commonly involved in dysregulation of 1) pathways in various cancers, 2) pathogen mediated diseases (Salmonella infection, measles, viral carcinogenesis, human Papillomavirus infection and Hepatitis B and C); 3) homeostatic cell regulation including p53, FoxO and mTOR signalling pathways, cell cycle, cell senescence, autophagy, and pathways regulating pluripotency of stem cells, 4) phosphatidylinositol and insulin signalling pathways and 5) cardiovascular hemodynamics (fluid shear stress and atherosclerosis) (Figure 4C).

Lastly, we extended our analysis to determine potential diseases that hsa‐miR‐4732‐3p may be involved. The OMIM database suggests this miRNA may be involved in various hematopoietic and solid cancers (27%), endocrine/metabolic disorders (27%), and neurodegenerative disorders (17%) (Figure 4D). Other diseases miR‐4732‐3p may be involved in include musculoskeletal (arthrogryposis, Paget disease), cardiovascular (arrhythmogenic right ventricular dysplasia), and dermatologic disorders (keratosis, nevus), among others (Figure 4D and Data S2).

In conclusion, our data suggest that short LEO spaceflight induced significant changes in plasma‐derived sEV miRNA content and identified hsa‐miR‐4732‐3p to be significantly upregulated post‐flight. Curiously, NASA's Twin Study showed an increase in carotid intima‐media thickness which remained stable 4 days post‐flight,^3^ whereas our bioinformatics analysis revealed hsa‐miR‐4732‐3p involvement in cardiovascular hemodynamics. Further, miR‐4732‐3p is upregulated in mesenchymal stromal cell‐derived sEVs following oxygen‐glucose deprivation and contributes to cardioprotection via reduction of apoptosis and levels of reactive oxygen species.^10^ Further longitudinal studies using samples from a larger astronaut cohort with paired clinical data are warranted to validate the utility of miR‐4732‐3p as a potential biomarker for monitoring astronauts’ health.

## CONFLICT OF INTEREST

The authors report no conflict of interest.

## Supporting information



Supplemental Figure 1. Characterization of small RNA sequencing data.

Supplement Material

Supplement Material

Supplement Material

## References

[1] Bezdan D , Grigorev K , Meydan C , et al. Cell‐free DNA (cfDNA) and exosome profiling from a year‐long human spaceflight reveals circulating biomarkers. iScience. 2020;23:101844. 33376973 10.1016/j.isci.2020.101844 PMC7756145

[2] Elgart SR , Little MP , Chappell LJ , et al. Radiation exposure and mortality from cardiovascular disease and cancer in early NASA astronauts. Sci Rep. 2018;8:8480. 29855508 10.1038/s41598-018-25467-9 PMC5981602

[3] Garrett‐Bakelman FE , Darshi M , Green SJ , et al. The NASA twins study: a multidimensional analysis of a year‐long human spaceflight. Science. 2019;364. 10.1126/science.aau8650 PMC7580864 30975860

[4] Pierson DL , Stowe RP , Phillips TM , Lugg DJ , Mehta SK . Epstein‐Barr virus shedding by astronauts during space flight. Brain Behav Immun. 2005;19:235‐242. 15797312 10.1016/j.bbi.2004.08.001

[5] da Silveira WA , Fazelinia H , Rosenthal SB , et al. Comprehensive multi‐omics analysis reveals mitochondrial stress as a central biological hub for spaceflight impact. Cell. 2020;183:1185‐1201.e1120. 33242417 10.1016/j.cell.2020.11.002 PMC7870178

[6] Bisserier M , Shanmughapriya S , Rai AK , et al. Cell‐Free mitochondrial DNA as a potential biomarker for astronauts' health. J Am Heart Assoc. 2021:e022055. 10.1161/JAHA.121.022055 34666498 PMC8751818

[7] Gertz ML , Chin CR , Tomoiaga D , et al. Multi‐omic, single‐cell, and biochemical profiles of astronauts guide pharmacological strategies for returning to gravity. Cell Rep. 2020;33:108429. 33242408 10.1016/j.celrep.2020.108429 PMC9444344

[8] Malkani S , Chin CR , Cekanaviciute E , et al. Circulating miRNA spaceflight signature reveals targets for countermeasure development. Cell Rep. 2020;33:108448. 33242410 10.1016/j.celrep.2020.108448 PMC8441986

[9] Garikipati VNS , Shoja‐Taheri F , Davis ME , Kishore R . Extracellular vesicles and the application of system biology and computational modeling in cardiac repair. Circulation Research. 2018;123:188‐204. 29976687 10.1161/CIRCRESAHA.117.311215 PMC6512976

[10] Sanchez‐Sanchez R , Martínez‐López V , Luján‐Juárez IA , et al. miR‐4732‐3p in extracellular vesicles from mesenchymal stromal cells is cardioprotective during myocardial ischemia. Front Cell Dev Biol. 2021;9:734143. 34532322 10.3389/fcell.2021.734143 PMC8439391

