# Supplementary material for: Space flight associated changes in astronauts’ plasma‐derived small extracellular vesicle microRNA: Biomarker identification
Source: Clin Transl Med. 2022 Jun 2;12(6):e845. doi: 10.1002/ctm2.845 (PMC9162436; doi:10.1002/ctm2.845)
Supplement: Supplementary file 1 — Supplemental Figure 1. Characterization of small RNA sequencing data. [file CTM2-12-e845-s004.docx]

**
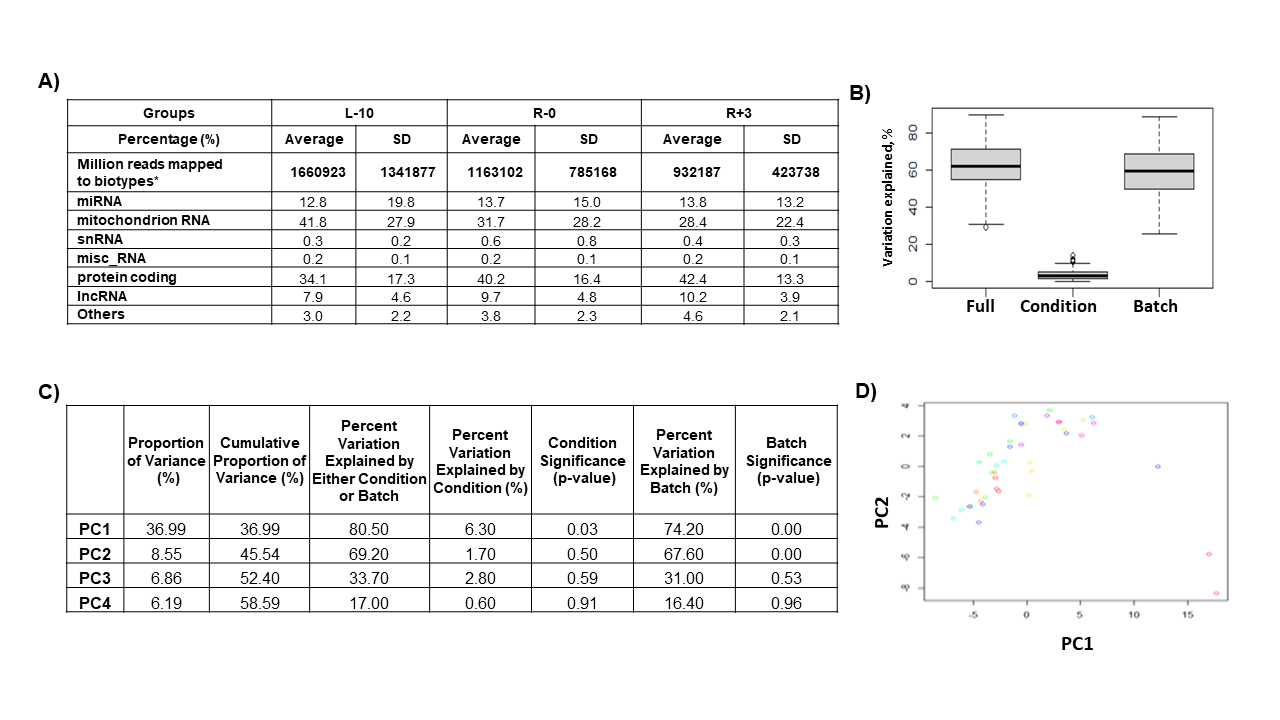
**

**Supplemental Figure 1. *Characterization of small RNA sequencing data.*** **(A)** Characterization of RNA sequencing data for all identified RNA types, including miRNA, mitochondrial RNA (mtDNA), small nucleolar RNA (snRNA), long non-coding RNA (lncRNA), miscellaneous RNA. **(B)** Variation analysis of the percentage of variation explained by the full model, condition (time-points), and batch (astronauts) across miRNA dataset. The results show that batch accounts for a considerable proportion of data variation. **(C)** Table of the percentage variation explained by batch and condition combinations for each of the top principal components in PCA analysis. **(D)** PCA plot of the first two components is shown.
