# Supplementary material for: Space flight associated changes in astronauts’ plasma‐derived small extracellular vesicle microRNA: Biomarker identification
Source: Clin Transl Med. 2022 Jun 2;12(6):e845. doi: 10.1002/ctm2.845 (PMC9162436; doi:10.1002/ctm2.845)
Supplement: Supplementary file 2 — Supplement Material [file CTM2-12-e845-s003.docx]

**Supplementary Information**

**Materials and Methods**

***Astronauts Samples.*** We studied changes in the sEV transcriptomic profile by small RNA sequencing using RNA isolated from PB plasma of 14 astronauts who flew median 12-day long space Shuttle missions between 1998-2001. Information regarding de-identified blood samples is limited to the crew's binned age in order to ensure privacy of involved personnel and is depicted graphically and in the table **(Figure 1A).** sEVs were isolated from PB plasma at three different time points: 10 days before launch (L-10), the day of landing (R-0), and three days after return (R+3). All samples were stored at -80°C until use. This study was approved by NASA and the Icahn School of Medicine at Mount Sinai's Institutional Review Board (STUDY00000075 and HSM19-00367, respectively). Blood samples were collected during a NASA flight study; astronauts provided written informed consent to participate in that study and have blood stored and used for residual analyses.

***Thrombin Plasma Preparation for sEVs Precipitation.*** sEVs were isolated from PB samples of 14 astronauts at L-10, R-0, and R+3 using the ExoQuick Plasma preparation and sEV precipitation kit (Cat # EXOQ5TM, System Biosciences, CA, USA). In brief, 400 μl of plasma was mixed with thrombin and kept at room temperature (RT) for 5 minutes. Subsequently, samples were centrifuged at 10,000 rpm for 5 minutes. Next, according to the manufacturer's protocol, the supernatant was collected into a new sterile microcentrifuge tube. Samples were then incubated with the exosome precipitation solution and refrigerated at 4°C for 30 minutes. After centrifugation at 1,500 x g for 30 minutes at 4°C, a beige-colored pellet was observed and dissolved in 100 μl of sterile 1xPBS.

**Nanoparticle Tracking Analysis (NTA).** NS-300 Nanosight instrument (Malvern Instruments Ltd., Malvern, UK) equipped with an sCMOS camera (Hamamatsu Photonics, Hamamatsu, Japan) and a 405 nm laser was used. Data acquisition and processing were performed using NTA software version 2.3 build 0025. Background extraction was applied, and automatic settings were employed to determine the minimum expected particle size, minimum track length, and blur settings. Data were obtained at camera level 12 (shutter: 600, gain: 350). Three movies of 30 seconds at 25 frames per second were recorded and assigned a single measurement in triplicates.

***Exosome Antibody Array.*** The exosome antibody array was performed using the EXO-check exosome antibody arrays (Cat # EXORAY210A-8, System Biosciences, CA, USA). Briefly, isolated sEVs were quantified for protein using the BCA assay kit (Cat # 23225, Thermo scientific, Il, USA.). Seventy five μg of protein were incubated with the labeling reagent for 30 minutes at RT. Excess labeling reagent was removed according to the manufacturer's protocol. Labeled exosomes were blocked using a blocking buffer, and the membrane was exposed with exosomes facing up at 4ºC overnight. The next day, the membrane was washed for 5 minutes at RT and incubated with the detection buffer for 30 minutes at RT. Subsequently, washing was done with wash buffer three times for 5 minutes and developed using the chemiluminescence detection system (Clarity Western ECL substrate, cat # 170-5060S, Bio-Rad, USA).

***Library Preparation and Small RNA Sequencing.*** RNA quality was assessed using an Agilent TapeStation (Agilent, Palo Alto, CA, USA), and RNA concentration was quantified by Qubit 4.0 spectrophotometer. The library for small RNA-sequencing (RNA-Seq) was prepared using the Smarter smRNAseq kit for Illumina (Takara Bio Inc., USA). The quantity and quality of amplified libraries were evaluated using Qubit (Invitrogen, Carlsbad, CA, USA) and Agilent TapeStation high sensitivity D1000 Screen Tape. Small RNA-seq libraries were sequenced using single-end 75 base pairs (PE75) sequencing chemistry on NextSeq 500 instruments following the manufacturer's protocols (Illumina).

***Sequencing Data Analysis.*** MiRNA sequencing data were pre-processed and analyzed using the miRDeep2 pipeline (1). The 3′ adaptor sequence was removed, and reads with the trimmed length <18 bp were discarded using Cutadapt v2.7 (2). Read quality control was performed with FastQC v0.11.9 http://www.bioinformatics.babraham.ac.uk/projects/fastqc/ Trimmed and filtered reads were mapped with bowtie (v1.1.1**)** (3) to the reference genome (GRCh38.p12), allowing a maximum of one mismatch. Calculation of the raw read counts and reads per million (RPM) values was performed using miRDeep2 (miRBase v22.0).

***Portraying Transcriptome Landscapes and Batch Effect Assessment.*** The overall variability of miRNA expression landscapes was assessed using the self-organizing map (SOM) machine learning approach with oposSOM R package (4). The SOM approach reduces the dimensionality of gene expression, clusters them into meta-genes on 40x40 grid, and visualizes deregulated clusters using maroon-red color gradients representing down- and up-regulated gene clusters in each sample, respectively (5). Batch effect analysis was performed using batchQC package for evaluating sample and batch effects in RNA-seq data (6).

***Differential Expression Analysis and Functional Annotation of miRNA expression.*** It has been suggested that different pipelines for differential expression analysis can produce different results depending on various factors, including sequencing depth, library preparation, and the number of biological replicates (7, 8). To ensure maximal reliability of detected differentially expressed genes (DEGs), we analyzed three pipelines to identify overlap of DEGs in all three pipeline analyses. First, we used the edgeR package (9) for library size normalization, dispersion correction, and differential expression analysis. Second, we used edgeR for library size normalization, dispersion correction in combination with limma for differential expression analysis (10). Finally, we used the RUVseq package (11) to estimate and remove unreliable variation (number of latent variables were set to 1) followed by edgeR for library size normalization, dispersion correction, and differential expression analysis. Individual astronaut information was included as a covariate in all models. MiRNAs with p values adjusted for FDR < 0.05 (fold change ± 1.4-3) were considered differentially expressed. Functional annotation of deregulated miRNAs was performed using the miRNA Enrichment Analysis and Annotation Tool (miEAA 2.0) tool (12) against Gene Ontology (GO), KEGG Pathway, Online Mendelian Inheritance in Man (OMIM), and other databases. Gene targets for differentially expressed miRNAs were obtained using the mirPathDB 2.0 database (12) and visualized with Cytoscape 3.9 (13).

***Real-Time Quantitative Reverse Transcription PCR.*** We isolated total RNA from sEVs isolated from PB samples of 7 astronauts (Z14, Z13, Z11, Z9, Z7, Z6, Z5) at L-10, R-0, and R+3 . According to the manufacturer's protocol, cDNA synthesis was performed with 5 ng of total RNA using the Taqman advanced miRNA cDNA synthesis kit (Cat # A28007, Applied Biosystem, USA). Real-time polymerase chain reaction (qRT-PCR) was performed using TaqMan probes (Master Mix, Cat #4444963, Applied Biosystem, USA) and QuantStudio^TM^ 3 Real-Time PCR systems as recommended by the manufacturer. We measured the expression of the following miRNAs indicated below.

| **Primer(s) used** | **Assay ID** |
| --- | --- |
| hsa-miR-92a-p | 477827_mir |
| hsa-miR-140-5p | 477909_mir |
| hsa-miR-26b-5p | 478418_mir |
| hsa-miR-363-3p | 478060_mir |
| hsa-miR-4732-3p | 478118_mir |
| hsa-miR-20b-5p | 477804_mir |
| hsa-miR-627-5p | 478427_mir |
| hsa-miR-483-5p | 478432_mir |
| hsa-miR-361-5p | 478056_mir |
| hsa-miR-26a-5p | 477995_mir |

***Statistical Analysis of qRT-PCR Results.*** Results are presented as mean ± standard error of the mean (SEM). Data were analyzed using an unpaired t-test for comparisons between means, 1-way analysis of variance with the Bonferroni correction for comparisons between >2 groups. Statistical analysis was performed using GraphPad Prism 9, version 9.2.0 (GraphPad Software, Inc., La Jolla, CA, USA). Differences were considered statistically significant at p < 0.05.

**Supplemental References**

1. M. R. Friedlander, S. D. Mackowiak, N. Li, W. Chen, N. Rajewsky, miRDeep2 accurately identifies known and hundreds of novel microRNA genes in seven animal clades. *Nucleic Acids Res* **40**, 37-52 (2012).

2. M. Martin, Cutadapt removes adapter sequences from high-throughput sequencing reads. *EMBnet.journal* **17**, 10-12 (2011).

3. B. Langmead, C. Trapnell, M. Pop, S. L. Salzberg, Ultrafast and memory-efficient alignment of short DNA sequences to the human genome. *Genome Biol* **10**, R25 (2009).

4. H. Loffler-Wirth, M. Kalcher, H. Binder, oposSOM: R-package for high-dimensional portraying of genome-wide expression landscapes on bioconductor. *Bioinformatics* **31**, 3225-3227 (2015).

5. H. Wirth, M. Loffler, M. von Bergen, H. Binder, Expression cartography of human tissues using self organizing maps. *BMC Bioinformatics* **12**, 306 (2011).

6. S. Manimaran *et al.*, BatchQC: interactive software for evaluating sample and batch effects in genomic data. *Bioinformatics* **32**, 3836-3838 (2016).

7. D. Liu *et al.*, MiR-361-5p acts as a tumor suppressor in prostate cancer by targeting signal transducer and activator of transcription-6 (STAT6). *Biochemical and biophysical research communications* **445**, 151-156 (2014).

8. Y. Su *et al.*, Circulating miR-19b-3p as a Novel Prognostic Biomarker for Acute Heart Failure. *J Am Heart Assoc* **10**, e022304 (2021).

9. M. D. Robinson, D. J. McCarthy, G. K. Smyth, edgeR: a Bioconductor package for differential expression analysis of digital gene expression data. *Bioinformatics* **26**, 139-140 (2010).

10. M. E. Ritchie *et al.*, limma powers differential expression analyses for RNA-sequencing and microarray studies. *Nucleic Acids Res* **43**, e47 (2015).

11. D. Risso, J. Ngai, T. P. Speed, S. Dudoit, Normalization of RNA-seq data using factor analysis of control genes or samples. *Nat Biotechnol* **32**, 896-902 (2014).

12. F. Kern *et al.*, miEAA 2.0: integrating multi-species microRNA enrichment analysis and workflow management systems. *Nucleic Acids Res* **48**, W521-W528 (2020).

13. P. Shannon *et al.*, Cytoscape: a software environment for integrated models of biomolecular interaction networks. *Genome Res* **13**, 2498-2504 (2003).
